# Data in question: A survey of European biobank professionals on ethical, legal and societal challenges of biobank research

**DOI:** 10.1371/journal.pone.0221496

**Published:** 2019-09-18

**Authors:** Melanie Goisauf, Gillian Martin, Heidi Beate Bentzen, Isabelle Budin-Ljøsne, Lars Ursin, Anna Durnová, Liis Leitsalu, Katharine Smith, Sara Casati, Marialuisa Lavitrano, Deborah Mascalzoni, Martin Boeckhout, Michaela Th. Mayrhofer

**Affiliations:** 1 BBMRI-ERIC, Graz, Austria; 2 Department of Sociology, University of Malta, Msida, Malta; 3 Norwegian Research Center for Computers and Law, Faculty of Law, University of Oslo, Oslo, Norway; 4 Centre for Medical Ethics, Faculty of Medicine, University of Oslo, Oslo, Norway; 5 Norwegian Institute of Public Health, Oslo, Norway; 6 Department of Public Health and Nursing, Norwegian University of Science and Technology, Trondheim, Norway; 7 Institute for Advanced Studies, Vienna, Austria; 8 Institute of Genomics, Estonian Genome Center, University of Tartu, Tartu, Estonia; 9 Centre for Health Policy, Institute of Global Health Innovation, Imperial College London, London, England, United Kingdom; 10 Department of Medicine and Surgery, University Milano—Bicocca, Milan, Italy; 11 Department of Public Health, Center for Research Ethics and Bioethics, University of Uppsala CRB, Uppsala, Sweden; 12 EURAC Research, Institute of Biomedicine, Bolzano, Italy; 13 Department of Medical Humanities, Julius Center for Health Sciences and Primary Care, University Medical Center Utrecht, Utrecht, The Netherlands; National Institute of Public Finance and Policy, INDIA

## Abstract

Biobanks have evolved, and their governance procedures have undergone important transformations. Our paper examines this issue by focusing on the perspective of the professionals working in management or scientific roles in research-based biobanks, who have an important impact on shaping these transformations. In particular, it highlights that recent advances in molecular medicine and genomic research have raised a range of ethical, legal and societal implications (ELSI) related to biobank-based research, impacting directly on regulations and local practices of informed consent (IC), private-public partnerships (PPPs), and engagement of participants. In our study, we investigate the ways that these concerns influence biobanking practices and assess the level of satisfaction of the cross-national biobanking research communities with the ELSI related procedures that are currently in place. We conducted an online survey among biobankers and researchers to investigate secondary use of data, informing and/or re-contacting participants, sharing of data with third parties from industry, participant engagement, and collaboration with industrial partners. Findings highlight the need for a more inclusive and transparent biobanking practice where biobanks are seen in a more active role in providing information and communicating with participants; the need to improve the current IC procedures and the role of biobanks in sharing of samples and data with industry partners and different countries, and the need for practical, tangible and hands-on ethical and legal guidance.

## Introduction

The European biobank landscape has experienced extensive growth and change over the last few decades [1–3]. Not only have the number of biobanks multiplied [1, 4] but the understanding of what constitutes a biobank has changed. Defined originally as mere ‘collections of samples and tissues’ [4] and ‘associated data’ [5–8], biobanks have evolved into complex infrastructures, which contribute in important ways to health research and operate at national, regional and global levels [9, 10]. This development, together with current advances in molecular medicine and genomics, has led to ethical, legal and societal implications (ELSI) related to three main areas of concern.

First, biobanks participants and stakeholders are in demand of greater transparency regarding the use and sharing of health data for research [11]. Transparency is required not only at the time of collection of the informed consent, but throughout the whole research process [12, 13]. There is increasing encouragement for feedback of research results to biobank participants [14, 15]. Transparency about the generation and managing of research data with possible health utility for research participants is vital in order for biobanks to develop ethically robust policies to cater for this facility.

Second, and related to the first, biobank participants, initially often viewed as simple ‘donors’ who allowed their sample to be used, are now becoming ‘participants’, ‘partners’, or even ‘stakeholders’ as a recognition of their active involvement in the biobank’s activities including research [2, 16]. There is growing understanding that *participant engagement* is essential in order to increase attention rates, maintain retention rates, build trust in the research, improve its relevance and utility, and enable continuous data collection to inform future research projects [17].

Third, publicly-funded biobanks are increasingly encouraged to develop *partnerships with industrial actors* such as pharmaceutical companies and biotechnology start-ups, to accelerate research discovery and promote the advancement of personalized medicine [18, 19]. Public-private partnerships (PPPs) impact established practices related to human biological specimens as well as health research-related data processing and sharing across biobanks and countries [19]. Studies show how collaborations with industry, envisioned to benefit science and society, must be further assessed in view of ELSI challenges such as conflicts of interests and questions around profit-based activities versus public health activities [18].

Many studies on biobanking have explored *public* attitudes towards ELSI challenges of biobanking [20–22] and have identified a need to involve participants in defining the consent process related to data processing [23], as well as a need to engage citizens in the related public discussion on biobanking [24]. Little has been done, however, to investigate the views of *professionals* in the field of biobanking regarding the ELSI challenges of biobanking. The needs related to biobank-based research have changed in the last past few years with increasing importance of ongoing of study participants, whether to contribute new data, to move into translational approaches or to support existing studies. The views of biobank professionals on participatory approaches play a major role in this respect and have not been closely explored to date. This paper contributes to fill this lacuna. Here, we report and discuss findings from a large online survey investigating the views of biobankers and researchers in relation to these three key issues.

Furthermore, the introduction of two recent major legal instruments has impacted directly on the issues under scrutiny here: 1) the European Union General Data Protection Regulation 2016/679 (GDPR) lays out new requirements in terms of *transparency*, *data subjects’ rights*, *and information about data uses* [25], and introduces the principles of accountability and direct responsibility of the processor towards the subject; 2) the Article 27 of The Additional Protocol to the Convention on Human Rights and Biomedicine, concerning Biomedical Research, establishes a *duty of care* in the countries that have ratified this Council of Europe instrument. Both these legal instruments have been shaping the ways the biobank community has understood its challenges and aims, and they create a core context to the interpretation of our survey, conducted just 10 weeks before GDPR and the Article 27 went into force. Although our survey was not designed as an analysis of implementation of GDPR, much less for making conclusions about the attitudes of biobankers towards this new and important legal landscape, the GDPR has indeed constituted the stage of reflection of current biobank governance. Viewed from that perspective, the GDPR impacts our survey by constituting an opportunity for research institutions and biobanks to adapt to these new legal frameworks, while laying out provisions and exceptions expressly thought of in the context of research.

Against this background, we asked biobankers and researchers to evaluate the issues of (1) participant information concerning enrolment, re-contact, and secondary use of data, (2) participant engagement, and (3) collaboration and sharing of data with industrial partners. This paper presents the experiences and opinions of 273 biobankers from 32 countries. After outlining our research design and methods, we discuss our data and flag resulting implications related to participant engagement and the everyday practice of biobanking.

This survey was conducted as a key task of BBMRI-ERIC, a research infrastructure for biobanks and biomolecular resources [26] that systematically scans the horizon for upcoming debates and questions surrounding ethical, legal, and societal aspects of biobanking, and provides professional support to biobanks through the Common Service ELSI [27] (http://www.bbmri-eric.eu/BBMRI-ERIC/about-elsi/) as well as the H2020 project ADOPT BBMRI-ERIC (http://www.bbmri-eric.eu/scientific-collaboration/adopt-bbmri-eric/), and the COST Action IS1303 CHIP ME (http://www.cost.eu/COST_Actions/isch/IS1303). Collaborators were the FP7 project RD-connect (https://rd-connect.eu), the IMI project DO-IT (https://www.imi.europa.eu/projects-results/project-factsheets/do-it) and Biobank Norway (https://www.ntnu.edu/biobanknorway). These projects and organizations have enabled discussions about biobank related issues across countries and disciplines.

## Methods

### Study design and participant recruitment

This study was driven by the key research question: how do the current changes related to the ethical, legal, societal and scientific landscape of biobanking impact on attitudes, beliefs and practices in the sector? Based on the hypothesis that ethical, legal and societal challenges, manifested in different local practices of IC, private-public partnerships, and engagement of participants are affecting the use of biobanks, we investigated both professional experiences and opinions about (1) secondary use and sharing of data and collaboration with industrial partners and (2) informing and/or re-contacting participants with a special focus on participant engagement.

The survey was sent to experts with experience as researchers or other professional activities related to biobanks and/or collections of biological samples. These inclusion criteria were confirmed in the survey. Along with the IC of respondents, these were necessary to start the online questionnaire. The link to the survey was disseminated primarily through the BBMRI-ERIC e-newsflash (approximately 6,000 subscribers), the BBMRI-ERIC Directory (https://directory.bbmri-eric.eu) (approximately 600 biobanks and collections), the BBMRI National Nodes networks, and the newsletters of the partners of this study. Associated organizations were also asked to further circulate the survey link. Additionally, the survey was promoted through various social media channels including @bbmrieric, @corbel, Twitter, and LinkedIn. Recruitment was carried out in two waves. First, the survey was widely promoted through the channels outlined previously. Second, recruitment was intensified in countries that were underrepresented one month before the survey closed. The survey was open between December 2017 and March 2018.

Data was collected via an online survey and administered using SurveyMonkey.com. Our research question was operationalised by collecting data on 116 items within 25 multiple choice questions. The survey was divided into four sections. The first collected information about the characteristics of the biobanks (type of collections, major source of funding, respondents’ position). The remaining questions were organized under three topics, where respondents were asked to provide answers regarding their experiences with, and opinions about IC, participant involvement, and public-private partnerships. Response options were derived from recent scientific literature discussed throughout the paper debating secondary use of data, informing and/or re-contacting participants, sharing of data with third parties from industry, participant engagement, and collaboration with industry partners. Items compiling details about what information is and should be provided in the IC procedure were selected in line with GDPR requirements. Open-ended answer fields were included to allow collection of more in-depth qualitative data. The questionnaire was pre-tested with potential respondents from the target group to evaluate comprehensiveness, length, and clarity, and was refined accordingly (see survey instrument in S1 File).

Data analysis focused on the following topics: (1) the content of the IC documents in use, identifying areas where the related IC procedure is considered by the biobankers using it to be lacking; (2) the state of play with regards to re-use of samples and associated re-consent, exploring attitudes on how these aspects should be managed within the IC document; (3) the level of data-linkage and data-sharing within respondents’ current biobanking practices; (4) the perceived need for professional legal and ethical support resources and (5) the state of play with regards to participant engagement and health industry stakeholder engagement.

Correlations were tested using the Pearson Chi-Square test in SPSS and values of p<0.05 were considered statistically significant. Responses to questions allowing open-ended answers were analysed thematically, in line with standards of qualitative research methodology. Responses were analysed for recurring topics as well as issues raised which were not covered by the given answer options. Themes arising from the answers were identified.

For legal data analysis purposes, questions concerning practices of and attitudes towards IC, participant involvement and public-private collaborations were divided into two groups depending on whether or not the respondent was based in a country where the GDPR applies. Country distribution was based the location of the biobank or research facility, as described by the respondent. Similarly, questions concerning return of results to research participants, were divided into three groups depending on the country’s relationship with the Additional Protocol to the Convention on Human Rights and Biomedicine concerning Biomedical Research (CETS No. 195).

### Ethics statement

Ethics approval for this survey was given by the Research Ethics Committee of the University of Milano–Bicocca. Electronic IC was given to study participants at the beginning of the online questionnaire. Respondents were informed about the research scope and type of research, that their answers would be confidential, and that the results of the survey would be presented in a way that avoids the identification of individual participants. Respondents gave their IC by ticking a checkbox to confirm that they understood the information provided to them and voluntarily agreed to participate in the survey. Re-contact for further studies and interviews would only be possible if the participants voluntarily provided contact details by following a link to a separate contact form that was not part of the survey.

## Results

### Respondents’ demographics

A total of 273 (263 from Europe, 10 from other countries) people completed the survey, although it was started by 400 people. This relatively high incompletion rate could be due to recipients believing, once beginning the survey, that they have insufficient knowledge of the IC procedure in the biobank or research facility in which they are based.

Respondents represent various roles and types of biobanks (see Table 1), and work across a total of 32 countries. Of these, the majority of respondents’ biobanks or research facilities are based in Italy (18.3%), followed by the Netherlands (13.9%), Switzerland (8.8%), and the United Kingdom (7.3%). Twelve countries are represented by a single respondent, 11 by between two and 10 respondents, and the remaining five are represented by between 11 and 19 respondents each. Well over half of respondents work in publicly financed biobanks, including both national and regional investments (59.6%), around a quarter of the biobanks are both publicly and privately financed (26.2%), while the remainder are privately financed (8.2%) or unknown by the respondent (17.6%).

**Table 1:** Respondents’ main role related to biobanks and types of biobanks.

Main role of respondent related to biobanks	n	%
Director/CEO/Manager	88	31.3%
Researcher	69	24.6%
Project manager	46	16.4%
ELSI consultant	33	11.7%
Administrative staff	27	9.6%
Clinician/Researcher	20	7.1%
Clinician	3	1.1%
Technical support	3	1.1%
Business and innovation support/consultant	3	1.1%
Genetic counsellor	1	0.4%
**Type of biobank referred to**		
Prospective disease-oriented biobank	146	51.6%
Biobank or tissue archive containing tissue/specimens leftover from health care interventions (‘residual use’)	124	43.8%
Population-based biobank (prospective population-based cohort)	99	35.0%
Biobank containing tissue/specimens leftover from specific research projects	98	34.6%
Genetic biobank	76	26.9%
Multi-purpose hospital-based biobank	5	1.8%
Biobank of veterinary resources	2	0.7%

The majority of respondents are biobankers or researchers from countries which adhere to the GDPR. These are Austria, Belgium, Bulgaria, Croatia, the Czech Republic, Denmark, Estonia, Finland, France, Germany, Greece, Hungary, Italy, Latvia, Malta, the Netherlands, Norway, Poland, Portugal, Spain, Sweden, and the United Kingdom. Respondents from countries where the GDPR does not apply include Antigua and Barbuda, Bolivia, Canada, Russia, Saint Barthelemy, Switzerland, Turkey, Ukraine, the United Arab Emirates, and the United States.

Respondents from the three subgroups that were built to analyse answers to questions concerning the return of results to research participants belonged to: (1) countries where the Additional Protocol to the Convention on Human Rights and Biomedicine concerning Biomedical Research is in force (Bulgaria, Hungary, Norway, Portugal, and Turkey), (2) countries which have signed, but not ratified the Additional Protocol (Denmark, Greece, Italy, Sweden, and the Ukraine), and (3) countries which have neither signed nor ratified the Additional Protocol (Antigua and Barbuda, Austria, Belgium, Bolivia, Canada, Croatia, the Czech Republic (note: the Czech Republic has signed, but not ratified the Additional Protocol after the survey had closed), Estonia, Finland, France, Germany, Latvia, Malta, the Netherlands, Poland, Russia, Saint Barthelemy, Spain, Switzerland, United Arab Emirates, the United Kingdom, and the United States).

### Informed consent

A key focus of the survey was to investigate the IC procedures in biobanks and biobank-based research. Needing samples and data for their research, respondents were asked both what information is provided by their IC, and what information they believe should be provided by the IC. Both sets of results are outlined in the following tables.

Table 2 lists 16 items which respondents assessed in terms of what ‘is provided’ and ‘is not provided’ in the current IC procedure in use in their organisation. Respondents were able to tick multiple responses.

**Table 2:** What is provided to participants/donors in respondents’ current IC procedures?

What information is provided to participants/donors in the IC procedure?		Is provided in the IC	Is not provided in the IC	I don’t know
General info about the biobank and who is responsible for the IC procedure	n	186	20	11
	%	85.7%	9.2%	5.1%
Contact details of the biobank	n	174	30	13
	%	80.2%	13.8%	6.0%
The purpose and (future) objectives of the associated research	n	191	16	8
	%	88.8%	7.4%	3.7%
Details about research conducted through the biobank (e.g. via an online tool)	n	72	124	16
	%	34.0%	58.5%	7.5%
Possibility for the participant/donor to be re-contacted for additional data/samples	n	135	66	13
	%	63.1%	30.8%	6.1%
Possibility of returning individual research results	n	129	68	14
	%	61.1%	32.2%	6.6%
Linkage of data/samples with data from other sources (e.g. registries, national statistics)	n	115	78	20
	%	54.0%	36.6%	9.4%
Sharing data/samples with other non-commercial research partners	n	165	33	18
	%	76.4%	15.3%	8.3%
Sharing data/samples with commercial and/or health industry partners	n	119	77	20
	%	55.1%	35.6%	9.3%
Sharing data/samples with parties in other EU countries	n	125	69	20
	%	58.4%	32.2%	9.3%
Sharing data/samples with parties in non-EU countries	n	110	82	23
	%	51.2%	38.1%	10.7%
Expected storage period for data/samples	n	119	81	13
	%	55.9%	38.0%	6.1%
The right to withdraw IC at any time and what happens to data and samples afterwards	n	192	13	9
	%	89.7%	6.1%	4.2%
Other rights of participants, e.g. right to access data or right to know how data is processed	n	118	75	20
	%	55.4%	35.2%	9.4%
The right to lodge a complaint with a supervisory authority (e.g. an ethics commission), including contact info	n	73	110	30
	%	34.3%	51.6%	14.1%

Survey results show that there are four items which are present in the vast majority of all ICs. This is consistent with what is expected, with 89.7% of participants including ‘the right to withdraw IC at any time, and what happens to data and samples afterwards’; 88.8% including ‘the purpose and (future) objectives of the associated research’, 85.7% including ‘general information of the biobank and who is responsible for the IC procedure’, and 80.2% including ‘the contact details of the biobank’.

The two items most frequently flagged as being absent from the current IC are ‘details about research conducted through the biobank (e.g. via an online tool)’, and the right to lodge a complaint with a supervisory authority (e.g. an ethics commission) including contact info’ with 58.5% and 51.6% of respondents indicating absence of these respectively. Interestingly, the latter was also the item that respondents were most unsure about, with 14.1% unable to indicate whether or not it was included in their IC, followed by ‘sharing data/samples with parties in non-EU countries’ with a rate of 10.7%. Table 2 shows a lack of compliance with the GDPR in some cases in that the consents are not always properly specified or informed.

One theme dominated the data collected in the ‘other’ free text field provided in the IC procedure. This was the issue of returns to the participant, with both financial compensation (none) and reimbursement (for reasonable out of pocket expenses) mentioned as being provided, as well as finer clarification on the ways incidental findings are returned, if severe and actionable. Some contributions fell under the theme of ‘active participants’, with ‘right to restrict access’, and ‘details of advantages and disadvantages of participating’ being mentioned. One respondent mentioned ‘consent is valid after death’ as an item that was included in their IC procedure, and another that ‘participation/non-participation will not affect their [medical] treatment’.

Table 3 shows how respondents assessed seven items in relation to what ‘should be provided’, and what ‘is not essential’ in the IC:

**Table 3:** What information should be provided to participants/donors in the IC?

In your opinion, what information should be provided to participants/donors in the IC?		Should be provided in the IC	Is not essential in the IC	I don’t know
The purpose and (future) objectives of the associated research	n	184	32	1
	%	84.8%	14.7%	0.5%
Possibility of re-contact by researchers for additional data/sampling	n	175	41	2
	%	80.3%	18.8%	0.9%
Possibility of returning individual research results	n	150	60	7
	%	69.1%	27.6%	3.2%
Sharing data/samples with other non-commercial research partners	n	194	17	4
	%	90.2%	7.9%	1.9%
Sharing data/samples with commercial and/or health industry partners	n	181	24	11
	%	83.8%	11.1%	5.1%
Sharing data/samples with parties in other EU countries	n	172	36	10
	%	78.9%	16.5%	4.6%
Sharing data/samples with parties in non-EU countries	n	169	39	11
	%	77.2%	17.8%	5.0%

All seven items were selected by at least 69.1% of the respondents. According to respondents, the top three items that ‘should be’ provided include ‘sharing data/samples with other non-commercial research partners’, flagged by 90.2% of respondents, ‘the purpose and (future) objectives of the associated research’ (84.8%), and ‘sharing data/samples with commercial and/or health industry partners’ (83.8%). The option least frequently selected is ‘possibility of returning individual research results’, chosen by 69% of respondents, with 27.6% stating that this is not essential for the IC.

There was no significant difference (p>0.05) between countries that do or do not apply the GDPR in terms of responses to questions about items currently covered by the IC, the information that should be covered in the IC, the type of consent currently in place, the possibility of data-linkage covered in the IC, practices and attitudes towards re-use of samples and data, participant involvement, and public-private partnerships.

Data from the two questions mentioned above, ‘what information is provided to participants/donors in the IC procedure?’ and ‘in your opinion, what information should be provided to participants/donors in the IC?’, were cross tabulated for the six items that coincide to assess respondents’ levels of satisfaction with the IC in use (Table 4). This analysis was limited to respondents who identified the items as missing from their current IC. Since respondents could select more than one option for both questions the percentages may exceed 100%. Out of the 136 participants who answered both, ‘is not provided’ and ‘should be provided’, 30.9% flag ‘sharing data/samples with parties in non-EU countries’ as being an issue that is not in their current IC, but should be. This was the most frequently flagged issue that respondents believed should be addressed. Other common issues include the ‘possibility for participant/donor to be re-contacted for additional data/samples’, flagged by 29.4% of respondents, and ‘sharing data/samples with parties in other EU countries’, flagged by 27.9%. The issue least frequently considered to be lacking, defined as one that respondents did not think ‘should be provided’, is ‘the purpose and (future) objectives of the associated research’. Only 2.2% of respondents indicated that it was not in their IC and that it ‘should be’ provided.

**Table 4:** Cross tabulation of 'not provided' with 'should be provided' in the IC.

			Should be provided						Total
			The purpose and (future) objectives of the associated research	Possibility for the participant/donor to be re-contacted for additional data/samples	Possibility of returning individual research results	Sharing data/samples with other non-commercial research partners	Sharing data/samples with parties in other EU countries	Sharing data/samples with parties in non-EU countries	
**Is not pro-vided**	The purpose and (future) objectives of the associated research	n	3	12	12	13	13	12	15
		%	2.2%	8.8%	8.8%	9.6%	9.6%	8.8%	
	Possibility for the participant/donor to be re-contacted for additional data/samples	n	48	40	40	54	50	47	64
		%	35.3%	29.4%	29.4%	39.7%	36.8%	34.6%	
	Possibility of returning individual research results	n	55	47	25	59	50	51	66
		%	40.4%	34.6%	18.4%	43.4%	36.8%	37.5%	
	Sharing data/samples with other non-commercial research partners	n	27	25	21	22	18	16	33
		%	19.9%	18.4%	15.4%	16.2%	13.2%	11.8%	
	Sharing data/samples with parties in other EU countries	n	61	51	43	55	38	37	68
		%	44.9%	37.5%	31.6%	40.4%	27.9%	27.2%	
	Sharing data/samples with parties in non-EU countries	n	72	61	51	66	46	42	81
		%	52.9%	44.9%	37.5%	48.5%	33.8%	30.9%	
**Total**		n	110	100	81	116	95	92	136

X^2^(25) = 29.78, p = 0.233

We compared the responses in the crosstabulation table on items that currently are not, but should be, covered in the IC with the responses in Table 2. There are noticeable differences in what is considered essential in IC. For instance, 84.8% of respondents consider ‘the purpose and (future) objectives of the associated research’ to be an important item to include in the IC. Yet, among those missing this item in their IC, only 2.2% think it should be added. Similarly, 69.1% stated that the ‘possibility of returning individual research results’ should be included in ICs, but only 18.4% of those who indicated that this item is missing in their IC stated that it should be included. This difference also exists for ‘sharing data/samples with other non-commercial research partners’, where 90.2% think this information should be provided in ICs while 16.2% stated that it is not included in their current IC, but should be.

When evaluating the IC procedure currently in use, 50.7% of the respondents stated that the IC they are using would benefit from some improvements, 43.0% think that the IC is sufficient and 6.3% didn’t know. 38 respondents elaborated on how they thought their IC would benefit from improvements. When analysed thematically, these contributions fell under three overarching themes: (1) information included in the IC; (2) clarity of text, and length of the document; and (3) a need for a dynamic, web-based component. Regarding the former, information flagged as lacking in the IC procedure reflect the topics discussed in previous questions. These include data linking, data sharing, future use of samples, and return of results (incidental findings). Two issues were new, however. One respondent highlighted the lack of choice of consent preferences and another the participant’s right to view their personal data. The latter issue will likely become more prominent as the GDPR raises awareness of participants’ rights and how to exercise them.

Document style and presentation was indicated as a source of dissatisfaction by four respondents. They highlighted unwieldy text, technical language and unrealistic length as issues requiring attention. Having a dynamic, web-based consent procedure was highlighted as a potential solution to inadequate active involvement by 10 participants. They listed advantages such as flexibility, getting updates on research progress, having an active choice about which research projects to be involved in, and the ease of withdrawing consent.

Regarding current IC practices, only 9.5% of respondents indicated that participants can choose their level of involvement (Table 5).

**Table 5:** Sample and data use currently covered by the biobanks’ IC.

Which sample and data use is currently covered by the IC in biobank you are referring to?	n	%
Data and samples will be used for multiple research projects without re-consent	126	59.7%
Data and samples will only be used for the study for which the participant gave consent	51	24.2%
Participants/donors can choose their consent preferences about what they want to be involved in (online or paper-based)	20	9.5%
Data can be used without consent because of a statutory exemption	8	3.8%
I don’t know	6	2.8%

Considering data re-use and re-consent. 50.5% of respondents stated that re-use should be practiced without re-consent, whereas 33.3% thought participants should be able to choose their preferences in terms of re-use of data and samples (Table 6).

**Table 6:** Opinions on how re-use should be practiced in biobanks.

In your opinion, how should re-use be practiced in biobanks?	n	%
Data and samples should be used for multiple research projects without re-consent	97	50.5%
Participants/donors should choose their consent preferences about what they want to be involved in	64	33.3%
Data and samples could be used without consent because they are anonymised	16	8.3%
Data and samples will only be used for the study for which the participant gave consent	15	7.8%

The type of biobank and role of respondent did not influence views about data re-use and reconsent (X^2^(24) = 25.81, p = 0.363). Nevertheless, the most frequent advocates of multiple uses of data without re-consent were directors/CEOs/managers of biobanks (64.9%) and project managers (59%). This said, re-consent is seen as necessary when not only the study, but also the research field changes. 44.4% of respondents stated that re-consent is necessary when data and samples are used for a study in a different field, whereas 34.4% consider re-consent unnecessary in that situation (Table 7).

**Table 7:** Opinions on re-consent.

In your opinion, when do you think re-consent is required?	n	%
If data/samples are to be used for a study in a different field	80	44.4%
I don’t think re-consent is necessary	62	34.4%
If new technology requires a change in practices	22	12.2%
If the field changes	13	7.2%
I don't know	3	1.7%

Thematic analysis of respondents’ additional comments emphasised significant change as an important factor. For instance, most respondents’ comments highlighted the importance of re-consenting only when there are significant changes related to the purpose of research or to participant-related implications. These could include participants’ level of involvement, a compromised level of anonymity, the possibility of returning findings due to the use of new technologies, or changes in biobank governance policies.

### Participant engagement

50.7% of respondents stated that there are no specific participant engagement activities undertaken by the biobank they were referring to. When in place, these activities mainly (38.3%) comprise information meetings (such as open days, public meetings, and science days). Respondents also defined public engagement as focus groups, surveys to participants, and web-based forums (Table 8).

**Table 8:** Biobank participants' involvement in activities of the biobank.

In which ways are biobank participants/donors involved in activities of the biobank?	n	%
No specific engagement activities in place	102	50.7%
Information meetings such as open days, public meetings, science days	77	38.3%
Focus groups or workshops to discuss specific aspects of the research	39	19.4%
Surveys to collect data about the participants’ perspectives on specific topics	38	18.9%
Open forums such as online, web-based participant forums	25	12.4%
**‎I don't know **	**7**	**3.5%**

Note: The total percentage exceeds 100% because the participants could select more than one option.

The main obstacles biobankers encounter for involving participants were too little time resources (51%) and too little financial resources (59.5%). Whereas only 17.5% of respondents indicated a lack of interest as an impediment to participant involvement (Table 9).

**Table 9:** Obstacles to participant involvement.

What obstacles for participant/donor involvement have you encountered in relation to your biobanking activities?	n	%
Too little financial resources	119	59.5%
Too little time resources	102	51.0%
No appropriate IT solutions for providing information	54	27.0%
Too little qualified staff	36	18.0%
No interest in involving participants	35	17.5%
Lack of information about findings gained by samples and data	32	16.0%
No obstacles encountered	11	5.5%
I don't know	**12**	**6.0%**

Note: The total percentage exceeds 100% because respondents could select more than one option.

This suggests that biobanks are seen to play an active role; not only as mere collections of samples and data, but as primary drivers for providing information and engaging with participants.

### Preferences for support

69.1% of respondents stated that when seeking information on how to design or improve their IC, they would seek ethical and legal guidance from professional information centres such as helpdesks. Additionally, 65.7% indicated that they would turn to national guidance or standards for IC and/or corresponding templates, 58.8% to international guidance of standards for IC and/or corresponding templates, and 41.2% to information from within their working environment or colleagues. The remaining respondents either search the internet (17.2%) or don’t know (2%) (Table 10).

**Table 10:** Preferences of information sources for designing and improving IC.

If you were designing/improving your IC, where would you search for information?	n	%
Ethical-legal guidance by professional information centres such as help desks for ethical and legal issues	141	69.1%
National guidance or standards for IC and/or corresponding templates	134	65.7%
International guidance or standards for IC and/or corresponding templates	120	58.8%
Information from within my working environment or ask colleagues	84	41.2%
The internet	35	17.2%
I don't know	**4**	**2.0%**

Note The total percentage exceeds 100% because the respondents could select more than one option.

These findings indicate that multiple sources of information are considered when designing or improving the IC. 17 other respondents shared additional considerations, such as the inclusion of key stakeholders like ethics committees, data protection officers, or patient advocacy groups.

Of the 207 respondents who answered this question, 73.9% indicated that they would use templates that follow national or international standards. 50.2% stated that web-based support such as interactive tools would be appreciated and 46.4% consider online information such as a web page (Table 11).

**Table 11:** Preferences for forms of professional ethical and legal support in relation to IC.

Which form/s of professional ethical and legal support in relation to the IC would you appreciate, if any?	n	%
Templates that follow national or international standards for IC	153	73.9%
Web-based support such as interactive tools	104	50.2%
Online information such as a web page	96	46.4%
Help desk	61	29.5%
Training	55	26.6%
I already have sufficient support	20	9.7%
Training or support are not necessary	4	1.9%
I don't know	**5**	**2.4%**

Note: The total percentage exceeds 100% because the respondents could select more than one option.

Additional comments received for this question hint towards a discrepancy between the number of publications available and the difficulty in translating the findings into practical solutions applicable to a local context.

### Public private partnerships

47.1% of respondents stated that their biobank has established collaborations with health industry partners, and 19.2% stated that there are plans to develop collaborations with stakeholders from the health industry. The results do not significantly differ between biobanks financed by public investments and biobanks financed by private investments (p>0.05). The remaining respondents answered either that their biobank had no collaboration with the industry (23.6%) or plans to collaborate in the future (1.9%) or that they did not know about any collaboration (8.2%) (Table 12).

**Table 12:** Collaboration between biobank and health industry partners.

Does your biobank collaborate with health industry partners?	n	%
Yes	98	47.1%
No	49	23.6%
Not yet, but we plan to develop collaborations with stakeholders from the health industry	40	19.2%
No, and we do not plan to do so	4	1.9%
I don’t know	17	8.2%

62.0% of respondents thought the biobank should explicitly ask for participants’ broad consent for collaborations with the health industry at the time of recruitment (Table 13). This is consistent with responses to the question about which information should be provided to participants/donors in the IC. The vast majority of respondents (83.8%) believed the IC should include a section to inform participants about the sharing of data and samples with commercial and health industry partners (Table 3). However, this does not always seem to be the case in practice, with 55.1% of respondents claiming that the sharing of data and samples with commercial and health industry partners is provided in the IC procedure.

**Table 13:** Preferences on how biobanks should inform research participants about potential or existing collaborations with stakeholders from the health industry.

In your opinion, how should biobanks inform their research participants/donors about potential or existing collaborations with stakeholders from the health industry?	n	%
Biobanks should explicitly ask for participants’ broad consent for collaborations with the health industry at the time of recruitment	124	62.0%
Biobanks should inform participants once such collaborations take place (e.g. newsletter or web page)	41	20.5%
Biobanks should ask for participants’ consent for each collaboration with the health industry	19	9.5%
Biobanks do not need to inform participants about collaborations with the health industry	16	8.0%

20.5% of respondents considered it sufficient to inform the participants about collaborations with industry partners through, for instance, a newsletter or web page (Table 13). A limited group of respondents (9.5%) deemed it necessary to collect the consent of participants each time a new collaboration is established with the health industry. Similarly, a limited group of respondents (8.0%) said biobanks do not need to provide information to participants about collaborations with the health industry. Further analysis showed, that there is a significant larger percentage of participants working in biobanks that collaborate with health industry partners (90.4%) who agree that sharing data with commercial and/or health industry partners should be provided in the IC, compared to their counterparts who work in biobanks that do not collaborate with health industry partners (72.9%). No significant differences in responses were found between publicly and privately funded biobanks.

Data show that respondents largely believe that collaboration with industry may bring a range of benefits including enabling the development of drugs and therapies (78.1%), increasing knowledge of disease and treatment (67.1%), and facilitating better research (57.1%). To a lesser extent, collaborations with health industry are expected to bring additional income to the biobank (41.0%), result in personal benefits for the biobank participants, for instance in the form of new drugs (12.9%), or lead to the creation of new jobs (10.0%).

There was, however, a concern among respondents that collaboration with the health industry may lead to increased focus on commercial profit rather than public health requirements (54.5%). This may translate to industrial partners determining the focus of the research (52.6%). Concerns were also raised about industrial partners earning money through a collaboration, while the biobank could get nothing equal in return (33.5%) or become dependent on private funding (26.3%). It should be noted that 20.6% of respondents considered none of the listed risks to be likely.

A large majority of respondents (86.4%) stated that solid contracts describing the responsibilities of the partners are important when collaborations are established with partners from the health industry. 52.9% believed that both parties in the collaboration should be well-aware of the details of the collaboration while 38.3% thought the partners in the collaboration should share data and samples as fairly as possible. Less emphasis was placed on the importance of fairly sharing benefits (35.9%) and risks (15%). Interestingly, 41.7% of respondents thought the details of the collaboration should be publicly available.

## Discussion

This study explores biobankers attitudes and the challenges they face in the context of evolving legal, ethical, societal and scientific landscapes related to biobanking. The survey data provide important insights into current practices and attitudes in this context and highlight the difficulties of responding to these changing landscapes. The survey findings provide a framework to foster debate about appropriate governance structures. This will improve IC procedures and public/participant engagement in biobanking.

### Challenges for informed consent

A key purpose of IC is to provide research participants with sufficient information to enable them to make an informed decision about the processing of personal data about them in biobanking. Transparency regarding data processing is not only a legal obligation given the GDPR, but also a preference of biobankers, as indicated in Table 3. Consent may also be used as a legal basis for data processing under the GDPR. 30.9% of respondents flagged that ‘sharing data/samples with parties in non-EU countries’ is not in their current IC but should be. If consent is used as a basis for transferring data to non-EU countries, the GDPR requires explicit consent to the transfer as well as the provision of information about the possible risks of such transfers. Research, especially given developments in genetics, has become heavily dependent on data sharing. Biobanks are encouraged to provide access to samples and data as well as to share data for secondary uses with national and international research consortia plus academic and/or industrial partners. The amount of information they provide to their participants about secondary use of data in the IC is nevertheless rather unexplored [28, 29]. Our findings show that informing participants about data sharing and multiple uses of data in biobank ICs is still not commonly practiced so far, despite being welcomed by biobankers, participants and the GDPR. In that respect, it is interesting that the inclusion of data-sharing with industrial partners as well as EU and non-EU countries in the IC is supported by the majority of our respondents when it comes to the different roles they have in biobanking, except for researchers. Our data hence suggests that while the importance of transparency related to data sharing in IC is not contested, further investigation is needed on why the opinions of researchers on data sharing in IC differ from other groups of respondents such as managers, administrative staff, ELSI consultants and project managers.

The legal requirements, as well as the ethical and societal aspects of biobanking, provide an impetus to use innovative communication formats. Viewed in this context, the IC procedure does more than accommodate legal requirements–it offers a complex integration of new requirements into the daily practices of biobanking, including the governance and organizational challenges this integration brings. Our data show that responses related to IC practices did not differ significantly between countries where the GDPR applies and where it does not.

Our data suggest that there is room for improvement. On one hand, respondents indicated that just over half of the items about the possibility that sample/data could be shared with different countries and stakeholders is included in the IC procedure (see Table 2). On the other hand, many respondents think such information should be provided in ICs (see Table 3). Findings from other quantitative and qualitative studies [5, 30, 31] that address data processing from the perspective of participants and the public, support providing information about data uses since concerns about data protection and potential use of personal data outside the research context influence preferences for consent types and participants’ views of biobank-based research.

The recent increase in data sharing, as well as demands to engage with participants in a more interactive way within and beyond IC procedures, raises the issue of how much information to provide to participants regarding potential recontact, feedback of results, and additional data collection. This challenge is also reflected in the apparent tension highlighted in the data between the need for more information in the consent document, and the perceived drawback of having long documents. Respondents’ preference for a form of web-based dynamic consent procedure offers a potential solution, particularly since electronic consent is explicitly mentioned as an option in the GDPR.

### Re-consent and return of results

Issues of re-consent and re-contacting participants are also linked to whether and how research findings should be returned to participants. Article 27 of The Additional Protocol to the Convention on Human Rights and Biomedicine, concerning Biomedical Research establishes a duty of care in the countries that have ratified this Council of Europe instrument, stating:

“If research gives rise to information of relevance to the current or future health or quality of life of research participants, this information must be offered to them. That shall be done within a framework of health care or counselling. In communication of such information, due care must be taken in order to protect confidentiality and to respect any wish of a participant not to receive such information.”

The Explanatory Report exemplifies the type of information that can be relevant to offer, mentioning “conclusions of the research or incidental information collected during the research” [32, paragraph 131]. It goes on to state that it is the researcher who must evaluate whether the information is of relevance to the current or future health or quality of life of research participants, and in doing so, the researcher may seek the advice of the ethics committee (Ibid.). The term “offered” has implications for the drafting of consent forms, as it requires that the wish of the participant to know or not to know is established prior to commencement of the research, including a participant's possible wish only to exercise their right to know under certain circumstances (Ibid. and paragraph 133. In certain circumstances, the right to know or not to know may be restricted on the basis of Article 26.1 of the Convention or in exceptional cases on the basis of domestic law in accordance with Article 10 of the Convention, see paragraph 134).

Although current legislation supports the return of individual research results and participants asking for them, the practice of biobanks toward this issue remains contested and ambivalent. Our data show that the option least frequently selected as information that should be provided in the IC is the ‘possibility of returning individual research results’, with 69% of participants responding with this option and 27.6% stating that it is not essential for the IC. There were no significant differences (X^2^(4) = 1.608, p = 0.807) in the views of the respondents based on whether or not they are located in a country that has signed the Additional Protocol to the Convention on Human Rights and Biomedicine. In each subgroup, approximately 70% of respondents stated that the ‘possibility of returning individual research results’ should be included in ICs. Interestingly, from 69.1% of respondents who indicated that return of results should be included in ICs, only 18.4% of those who indicated that this item is missing in their IC stated that it should be included.

This discrepancy raises feasibility issues and implies challenges on a structural level. A recent study among experts in Europe [33] identified a lack of legal frameworks, professional guidelines, and financial, organizational, and human resources as a key challenge for the return of individual genetic results to research participants. Clarifying legal requirements and harmonizing practices across Europe as well as cost-efficient IT-based platforms were proposed as possible solutions. How far these and other institutional challenges, such as lack of financial and time resources that were indicated as major obstacles in engaging with research participants, may also be an issue for returning results should be investigated. However, studies showed that research participants tend to be in favour of receiving research results [5, 34, 35]. One such study [35] showed, for instance, that most participants would be comfortable with inexpensive and easy-to-handle methods of returning information. Our findings regarding return of results echo a need to improve communication between biobanks and participants, which may also benefit engagement strategies used to inform participants.

Views on re-consent and satisfaction with the IC in use differ significantly between countries where the GDPR applies and where it does not. Re-consent is deemed necessary by respondents from GDPR countries if the data are to be used for a study in a different field (46.2% compared to 35.3% in non-GDPR countries). Respondents from countries where the GDPR does not apply are in favour of re-consent if new technology requires a change in practices (29.4% vs. 10.1%). Nevertheless, 36.1% of respondents from GDPR countries view re-consent as unnecessary. Questions around if and how biobanking participants should re-consent or be re-contacted as well as if and how research findings should be returned, is debated among ELSI scholars [33, 36]. Empirical studies have shown that most research participants want to have a say on how their samples and data are used in research [35, 37]. Participants tend to favour re-contact or re-consent when there are changes to the circumstances under which research with their materials and data is performed [29, 33]. These findings reflect the challenges around collecting samples and data for unknown purposes and uses in future research. The latter underlies biobankers’ preference for broader forms of IC [38]. This preference is also reflected in our survey. However, qualitative investigations have shown that preferences for broad consent are not made unconditionally by scientists as well as citizens and participants [5, 38]. In the present study, this becomes particularly apparent, demonstrated by a discrepancy between IC practices and attitudes of biobankers. For example, around one third of respondents stated that the information about sharing of data and samples within and outside the EU, and the possibility for participant to be re-contacted for additional data/samples, is not included in their current IC, but should be. So far, there is no consensus about the appropriate consent model among scientists [38], citizens/participants [5] and ELSI scholars [39]. Furthermore, broad consent is only allowed as an exception in cases where it is impossible to fully identify the purpose of the data processing at the time of data collection (see Recital 33 GDPR).

Various items in the survey indicate the importance of interacting with research participants and giving them a greater say over uses of their donated samples and data. 33.3% of respondents stated that a dynamic model should be practiced for re-use, 44.4% indicated that re-consent is necessary when data/samples are to be used for a study in a different field, and a majority of 62.0% of respondents think it is important to inform participants explicitly of collaborations with health industry at the time of recruitment to enable them to give IC about data and sample uses in such partnerships. A possible solution for these challenges could, in certain contexts, be found in web-based dynamic consent platforms [29, 40]. Such tools have been described and implemented to manage re-contact and more flexible ways of re-consenting, which may also support better engagement, especially for population-based biobanks. That said, engagement should not be limited to IC practices.

### Participant engagement

Engaging participants in the activities and governance of biobanks has become an important subject in debates about biobanks’ sustainability. Various engagement exercises have been developed and realized to explore public perceptions of and attitudes towards biobanks, and to tighten the relationship between biobanks and participants [5, 41, 42]. Typically, their aim is to deliberate about IC and its ethical, legal and societal implications, including the challenges mentioned in the introduction. Despite their goal to engage participants and other members of the public, these exercises have been criticized for their tendency to inform people about biobanks, rather than involve them in the activities around biobanking.

Our survey data confirm this trend as they show that participant engagement has not yet become a norm in many biobanks. Half of the respondents indicated that participants are not involved in biobank activities. When they are involved, this mainly comprises various forms of meetings where information is provided rather than engaged with. This result supports findings and conclusions of previous studies which concluded that the involvement of participants is practiced predominantly as upstream and one-way information provision [5, 43].

Our findings suggest a more inclusive and transparent biobanking practice as biobanks are seen to be playing a more active role in providing information and engaging with participants. This assumption is supported by respondents’ views about obstacles to participant engagement. Only 17.5% of respondents indicated that a lack of interest is impeding participant involvement. Engagement activities seem to be challenged by institutional or structural conditions, indicated in the survey data as too little time and resources. Whereas previous studies focused predominantly on the challenges of engagement models, the institutional and structural embeddedness of participant engagement are rather unexplored and should be subject to further investigation.

### Public-private partnerships

Policymakers strongly encourage the development of partnerships between biobanks and the health industry. Public-private partnerships (PPPs) are envisioned to bring new scientific discoveries and benefits to society such as medical applications and jobs [18]. While survey respondents largely acknowledge the potential scientific benefits of public-private partnerships, they expect PPPs to have a limited impact on economic growth, for instance through the creation of jobs. The potential economic benefits of PPPs are not fully documented and have been recently questioned [44]. This suggests that scientific needs and societal benefit, rather than economic ambitions, should be the main motivators in the establishment of PPPs [45].

Our data highlight the importance of empowering participants to make informed decisions about this issue. A majority of respondents indicated that it is important to inform participants explicitly of collaboration with health industry partners. Accordingly, participants should, at enrolment to studies or later, be able to base their consent on specific information about how their data and samples might be used in such collaborations. This is in line with results from recent studies, which show that participants are interested in having a say about the sharing of their data [37]. This leads to the question of whether broad consent, the approach preferred by our respondents, provides sufficient information about collaboration with industrial partners to be legally valid, or whether more specific forms of consent are needed not only for legal compliance, but to address the concerns and needs of research participants [46].

European citizens have been shown to be sceptical of sharing their data with the private sector [47, 48] and tend to perceive research carried out by private actors as primarily profit-oriented [5]. Increased transparency may contribute to raising awareness and reflection among participants regarding the potential advantages (and drawbacks) of establishing collaborations with industrial actors. Transparency about the terms of the collaboration will also be central when reconciling two groups of researchers–public and private–following different norms and objectives [49]. Guidelines and strategies are under development aiming to help biobank researchers establish PPPs in ways that protect the interests of the biobanks and the participants while promoting public health [50, 51].

## Conclusions

As our survey shows, those who take up diverse managing roles in current biobanking see both the necessity and the difficulty related to adapting their practices to changes in the ethical, legal, societal and scientific landscape of biobanks. In particular, further investigations on how transparency can be increased within IC in terms of providing more concrete information on data sharing with industrial partners is needed. Our survey sets a stage for such investigations as it went beyond the mere observation of these difficulties, to allow a stronger focus on structures that are and/or should be provided to ensure such integration. In this context, our findings also tune in to the call for practical solutions to improve IC, especially regarding cooperation, support, communication, engagement and new legal requirements. The lack of resources for such improvement–including time, financial, or human resources–is experienced across European countries despite their diversity in legal and institutional situations. At the same time, no matter how detailed and extensive a template of IC might be, biobankers must adapt them for their own specific context. They must embed engagement activities into their organisation’s clinical and research practices.

In that context, while it is important to continuously and systematically generate awareness among biobankers about ELSI aspects of their work, the responses in our survey demonstrate that identifying appropriate institutional structures is key to address the current challenges and requirements of research. Our data show that respondents seek ethical and legal guidance from professional information centers, turn to national and international guidance for IC or opt for templates that follow national and international standards as the most appreciated form of support. They particularly express the difficulty in translating these templates into practical solutions.

Future investigations should thus focus on translational and governance structures of biobanking, which take the local and organisational setting of a biobank into account. Related challenges were expressed by respondents as concerns about how to assess increasing participant involvement while maintaining biobanks’ ability to function well. The lack of translation structures is the main obstacle to advance patient engagement and bring an institutional routine to public engagement formats. Increased transparency and new modes of IC could motivate people to support collaborations with industry and open up a novel research area of governance in biobanking. This is particularly topical given the legal, ethical and societal requirements of the recently adopted GDPR. Our findings offer a starting point for conversations about where to place the focus of future biobank governance, and how to conceptualize upcoming research related to the translation of new requirements into biobanks’ institutional routines.

## Limitations

Our survey was conducted online, which might exclude respondents who are less familiar or comfortable with this method. There is also a sight asymmetry in the countries represented in the survey with larger percentages of respondents from Italy and the Netherlands.

This survey was conducted shortly before the EU GDPR became applicable but after it was adopted. It is therefore likely that the respondents’ organisations were already working on compliance with the GDPR.

## Supporting information

S1 File Questionnaire: “Data in question. ELSI Challenges in Biobank-based Research”. The online questionnaire as it appeared on the website/host SurveyMonkey.
(PDF)

## References

[1] Caenazzo L, Tozzo P. Biobanks and Public Health: A New Challenge for Public Engagement and Trust. Journal of Biomedical and Clinical Research. 2016;9(1):17–20.

[2] Gottweis H, Gaskell G, Starkbaum J. Connecting the public with biobank research: reciprocity matters. Nature Reviews Genetics. 2011;12(11):738–9. 10.1038/nrg3083 22005975

[3] Liu A, Pollard K. Biobanking for personalized medicine In: Karimi-Busheri F, editor. Biobanking in the 21st Century Advances in Experimental Medicine and Biology. Heidelberg, New York, Dordrecht, London: Springer, Cham; 2015 p. 55–68. 10.1007/978-3-319-20579-3_5 26420613

[4] Spjuth O, Krestyaninova M, Hastings J, Shen H-Y, Heikkinen J, Waldenberger M, et al Harmonising and linking biomedical and clinical data across disparate data archives to enable integrative cross-biobank research. Eur J Hum Genet. 2016;24(4):521–8. 10.1038/ejhg.2015.165 26306643 PMC4929882

[5] Goisauf M, Durnová A. From Engaging Publics to Engaging Knowledges: Enacting “Appropriateness” in the Austrian Biobank Infrastructure. Public Underst Sci. 2018;28(3):275–89. 10.1177/0963662518806451 30324869

[6] Murad AM, Myers MF, Thompson SD, Fisher R, Antommaria AHM. A qualitative study of adolescents’ understanding of biobanks and their attitudes toward participation, re‐contact, and data sharing. American Journal of Medical Genetics Part A. 2017;173(4):930–7. 10.1002/ajmg.a.38114 28328120

[7] Cambon-Thomsen A. The social and ethical issues of post-genomic human biobanks. Nature Reviews Genetics. 2004;5:866–73. 10.1038/nrg1473 15520796

[8] Mayrhofer MT. About the New Significance and the Contingent Meaning of Biological Material and Data in Biobanks. History and Philosophy of the Life Sciences. 2013;35(3):449–67. 24779112

[9] Harris JR, Burton P, Knoppers BM, Lindpaintner K, Bledsoe M, Brookes AJ, et al Toward a roadmap in global biobanking for health. European Journal of Human Genetics. 2012;20(11):1105–11. 10.1038/ejhg.2012.96 22713808 PMC3477856

[10] Mayrhofer MT, Prainsack B. Being a member of the club: The transnational self-governance of networks of biobanks. International Journal of Risk Assessment and Management 2009;12(1):64–81. 10.1504/IJRAM.2009.024130

[11] Lemke AA, Wolf WA, Hebert-Beirne J, Smith ME. Public and biobank participant attitudes toward genetic research participation and data sharing. Public Health Genomics. 2010;13(6):368–77. 10.1159/000276767 20805700 PMC2951726

[12] Murphy J, Scott J, Kaufman D, Geller G, LeRoy L, Hudson K. Public perspectives on informed consent for biobanking. American Journal of Public Health. 2009;99(12):2128–34. 10.2105/AJPH.2008.157099 19833988 PMC2775766

[13] D’Abramo F, Schildmann J, Vollmann J. Research participants’ perceptions and views on consent for biobank research: a review of empirical data and ethical analysis. BMC medical ethics. 2015;16(1):60 10.1186/s12910-015-0053-5 26354520 PMC4563851

[14] Thorogood A, Dalpé G, Knoppers BM. Return of individual genomic research results: are laws and policies keeping step? European Journal of Human Genetics. 2019:1 10.1038/s41431-018-0311-3. PMC6460582 30622328

[15] Bredenoord AL, Onland‐Moret NC, Van Delden JJ. Feedback of individual genetic results to research participants: in favor of a qualified disclosure policy. Human mutation. 2011;32(8):861–7. 10.1002/humu.21518 21538687

[16] Lunshof JE, Church GM, Prainsack B. Raw Personal Data: Providing Access. Science. 2014;343(6169):373–4. 10.1126/science.1249382 24458627

[17] Concannon TW, Fuster M, Saunders T, Patel K, Wong JB, Leslie LK, et al A systematic review of stakeholder engagement in comparative effectiveness and patient-centered outcomes research. Journal of general internal medicine. 2014;29(12):1692–701. 10.1007/s11606-014-2878-x 24893581 PMC4242886

[18] Hofman P, Bréchot C, Zatloukal K, Dagher G, Clément B. Public–private relationships in biobanking: a still underestimated key component of open innovation. Virchows Archiv. 2014;464(1):3–9. 10.1007/s00428-013-1524-z. 10.1007/s00428-013-1524-z 24337181

[19] Hämäläinen I, Toernwall O, Simell B, Zatloukal K, Perola M, van Ommen G-JB. Role of Academic Biobanks in Public–Private Partnerships in the European Biobanking and BioMolecular Resources Research Infrastructure Community. Biopreservation and Biobanking. 2018;17(1). 10.1089/bio.2018.0024 30499696

[20] Lipworth W, Forsyth R, Kerridge I. Tissue donation to biobanks: a review of sociological studies. Sociology of health & illness. 2011;33(5):792–811. 10.1111/j.1467-9566.2011.01342.x 21592141

[21] Sanderson SC, Brothers KB, Mercaldo ND, Clayton EW, Antommaria AHM, Aufox SA, et al Public attitudes toward consent and data sharing in biobank research: a large multi-site experimental survey in the US. The American Journal of Human Genetics. 2017;100(3):414–27. 10.1016/j.ajhg.2017.01.021. 10.1016/j.ajhg.2017.01.021 28190457 PMC5339111

[22] Critchley C, Nicol D, McWhirter R. Identifying public expectations of genetic biobanks. Public Underst Sci. 2016;26(6):1–17. 10.1177/0963662515623925 26769748

[23] McCormack P, Kole A, Gainotti S, Mascalzoni D, Molster C, Lochmü H, et al ‘You should at least ask’. The expectations, hopes and fears of rare disease patients on large-scale data and biomaterial sharing for genomics research. European Journal of Human Genetics. 2016;24:1403–8. 10.1038/ejhg.2016.30 27049302 PMC5027679

[24] Hoeyer K. The power of ethics: a case study from Sweden on the social life of moral concerns in policy processes. Sociology of Health & Illness. 2006;28(6):785–801. 10.1111/j.1467-9566.2006.00542.x 17184418

[25] Regulation (EU) 2016/679 of the European Parliament and of the Council of 27 April 2016 on the protection of natural persons with regard to the processing of personal data and on the free movement of such data, and repealing Directive 95/46/EC (General Data Protection Regulation), (2016).

[26] Mayrhofer MT, Holub P, Wutte A, Litton J-E. BBMRI-ERIC: the novel gateway to biobanks. Bundesgesundheitsblatt—Gesundheitsforschung—Gesundheitsschutz. 2016;59(3):379–84. 10.1007/s00103-015-2301-8 26860601

[27] Mayrhofer MT, Schlünder I. Mind the Gap: From Tool to Knowledge Base. Biopreservation and Biobanking. 2018;16(6):458–62. 10.1089/bio.2018.0018 30102551 PMC6308279

[28] Burton PR, Banner N, Elliot MJ, Knoppers BM, Banks J. Policies and strategies to facilitate secondary use of research data in the health sciences. International Journal of Epidemiology. 2017;46(6):1729–33. 10.1093/ije/dyx195 29025140 PMC5837447

[29] Kaye J, Whitley EA, Lund D, Morrison M, Teare H, Melham K. Dynamic consent: a patient interface for twenty-first century research networks. European Journal of Human Genetics. 2015;23:141–6. 10.1038/ejhg.2014.71 24801761 PMC4130658

[30] Joly Y, Dalpé G, So D, Birko S. Fair Shares and Sharing Fairly: A Survey of Public Views on Open Science, Informed Consent and Participatory Research in Biobanking. PLoS ONE. 2015;10(7). 10.1371/journal.pone.0129893. PMC4495996 26154134

[31] Petersen I, Desmedt C, Harris A, Buffa F, Kollek R. Informed consent, biobank research, and locality: perceptions of breast cancer patients in three European countries. Journal of Empirical Research on Human Research Ethics. 2014;9(3):48–55. 10.1177/1556264614540600 25746784

[32] Explanatory Report–CETS 195 –to the Additional Protocol to the Convention on Human Rights and Biomedicine, concerning Biomedical Research, (2005).

[33] Budin-Ljøsne I, Mascalzoni D, Soini S, Machado H, Kaye J, Bentzen HB, et al Feedback of Individual Genetic Results to Research Participants: Is It Feasible in Europe? Biopreservation and Biobanking. 2016;14(3):241–8. 10.1089/bio.2015.0115 27082461 PMC4913503

[34] Ormond KE, Smith ME, Wolf WA. The Views of Participants in DNA Biobanks. Stanford Journal of Law, Science & Policy. 2010;1:80–7.

[35] Mester JL, Mercer M, Goldenberg A, Moore RA, Eng C, Sharp RR. Communicating with Biobank Participants: Preferences for Receiving and Providing Updates to Researchers. Cancer Epidemiology Biomarkers & Prevention. 2015;24(4):708–12. 10.1158/1055-9965.Epi-13-1375 25597748 PMC4383666

[36] Steinsbekk KS, Solberg B. Biobanks—When is Re-consent Necessary? Public Health Ethics. 2011;4(3):236–50. 10.1093/phe/phr031.

[37] Hemminki E, Tupasela A, Jallinoja P, Aro AR, Snell K, Sihvo S. Finnish people's attitudes towards biomedical research and its sponsorship. Genomics, Society and Policy. 2009;5(2):67–79. 10.1186/1746-5354-5-2-67.

[38] Master Z, Campo-Engelstein L, Caulfield T. Scientists’ perspectives on consent in the context of biobanking research. European Journal of Human Genetics. 2015;23:569–74. 10.1038/ejhg.2014.143. 10.1038/ejhg.2014.143 25074466 PMC4402622

[39] Caulfield T, Murdoch B. Genes, cells, and biobanks: Yes, there’s still a consent problem. PLoS Biol. 2017;15(7). 10.1371/journal.pbio.2002654. PMC5526496 28742850

[40] Teare HJA, Hogg J, Kaye J, Luqmani R, Rush E, Turner A, et al The RUDY study: using digital technologies to enable a research partnership. European Journal of Human Genetics. 2017;25:816–22. 10.1038/ejhg.2017.57 28443622 PMC5520069

[41] Secko DM, Preto N, Niemayer S, Burgess M. Informed consent in biobank research: A deliberative approach to the debate. Soc Sci Med. 2009;68(4):781–9. 10.1016/j.socscimed.2008.11.020. 10.1016/j.socscimed.2008.11.020 19095337

[42] Burgess MM. From ‘trust us’ to participatory governance: Deliberative publics and science policy. Public Underst Sci. 2014;23(1):48–52. 10.1177/0963662512472160. 10.1177/0963662512472160 24434712

[43] Felt U, Fochler M. Machineries for Making Publics: Inscribing and De-scribing Publics in Public Engagement. Minerva. 2010;48(3):219–38. 10.1007/s11024-010-9155-x

[44] Rensch S. EU public private partnerships not economically viable, say auditors. Public Finance International [Internet]. 2018 10.10.2018. Available from: https://www.publicfinanceinternational.org/news/2018/03/eu-public-private-partnerships-not-economically-viable-say-auditors.

[45] Steinsbekk KS, Ursin LØ, Skolbekken J-A, Solberg B. We’re not in it for the money—lay people’s moral intuitions on commercial use of ‘their’biobank. Medicine, Health Care and Philosophy. 2013;16(2):151–62. 10.1007/s11019-011-9353-9 22028241 PMC3617351

[46] Caulfield T, Burningham S, Joly Y, Master Z, Shabani M, Borry P, et al A review of the key issues associated with the commercialization of biobanks. Journal of Law and the Biosciences. 2014;1(1):94–110. 10.1093/jlb/lst004. 10.1093/jlb/lst004 27774156 PMC5033518

[47] Special Eurobarometer 460. Attitudes towards the impact of digitisation and automation on daily life. European Commission, 2017.

[48] Ipsos MORI. The one-way mirror: public attitudes to commercial access to health data. Report prepared for the Wellcome Trust. 2016.

[49] Aarden E. Projecting and producing ‘usefulness’ of biomedical research infrastructures; or why the Singapore Tissue Network closed. Science and Public Policy. 2017;44(6):753–62. 10.1093/scipol/scx010

[50] Tierney WM, Meslin EM, Kroenke K. Industry Support of Medical Research: Important Opportunity or Treacherous Pitfall? Journal of General Internal Medicine. 2016;31(2):228–33. 10.1007/s11606-015-3495-z 26307387 PMC4720652

[51] Meslin EM, Rager JB, Schwartz PH, Quaid KA, Gaffney MM, Duke J, et al Benchmarks for ethically credible partnerships between industry and academic health centers: beyond disclosure of financial conflicts of interest. Clinical and Translational Medicine. 2015;4(1):36 10.1186/s40169-015-0077-y 26668063 PMC4678144

