# Correction: Data in question: A survey of European biobank professionals on ethical, legal and societal challenges of biobank research

**DOI:** 10.1371/journal.pone.0226149

**Published:** 2019-12-02

**Authors:** Melanie Goisauf, Gillian Martin, Heidi Beate Bentzen, Isabelle Budin-Ljøsne, Lars Ursin, Anna Durnová, Liis Leitsalu, Katharine Smith, Sara Casati, Marialuisa Lavitrano, Deborah Mascalzoni, Martin Boeckhout, Michaela Th Mayrhofer

In the Introduction, there are several errors in the sixth paragraph. The correct paragraph is:

Furthermore, two legal instruments has impacted directly on the issues under scrutiny here: 1) the European Union General Data Protection Regulation 2016/679 (GDPR) lays out new requirements in terms of *data subjects’ rights and information about data uses* [25], and introduces the principles of transparency and accountability; 2) the Article 27 of The Additional Protocol to the Convention on Human Rights and Biomedicine, concerning Biomedical Research, establishes a *duty of care* in the countries that have ratified this Council of Europe instrument. Both these legal instruments have been shaping the ways the biobank community has understood its challenges and aims, and they create a core context to the interpretation of our survey, conducted just 10 weeks before GDPR went into force. Although our survey was not designed as an analysis of implementation of GDPR, much less for making conclusions about the attitudes of biobankers towards this new and important legal landscape, the GDPR has indeed constituted the stage of reflection of current biobank governance. Viewed from that perspective, the GDPR impacts our survey by constituting an opportunity for research institutions and biobanks to adapt to this new legal framework, while laying out provisions and exceptions expressly thought of in the context of research.
