# Supplementary material for: Data in question: A survey of European biobank professionals on ethical, legal and societal challenges of biobank research
Source: PLoS One. 2019 Sep 18;14(9):e0221496. doi: 10.1371/journal.pone.0221496 (PMC6750647; doi:10.1371/journal.pone.0221496)
Supplement: S1 File — The online questionnaire as it appeared on the website/host SurveyMonkey. (PDF) [file pone.0221496.s001.pdf]

BBMRI-ERIC / COST ACTION CHIP ME / RD-CONNECT / IMI DO-IT / BIOBANK NORWAY

1. Welcome to the Survey: Data in Question. ELSI Challenges in biobank-based research

Thank you for participating in our survey.

It consists of 25 questions on some of the challenges of obtaining and sharing biological samples and data for research purposes in biobanks. This will help us to understand some of the challenges in this field that are due to new practices, technologies and changes in European law. The results of this survey will inform the community and may later be used to inform the development of policy. This survey is part of a research study being conducted by BBMRI-ERIC, COST Action CHIP ME, RD-Connect and IMI DO-IT, with contributions from Biobank Norway. For further information about the survey click here.

Your responses will be confidential and the results of the survey will be presented in a way that will not allow the identification of individual participants.

We would be grateful if we could contact you directly for further research purposes. If you agree please provide your contact details here. You can also contact us by sending an email to: contact@bbmri-eric.eu

V03.08.2017

* 1. Electronic Consent

- ○ I have read the above information and voluntarily agree to participate in this survey.
- ○ I disagree and do not wish to participate in the research study.

* 2. Do you have experience with research or other professional acitivities that are related to biobanks and/or collections of biological samples?

- ○ Yes
- ○ No

BBMRI-ERIC / COST ACTION CHIP ME / RD-CONNECT / IMI DO-IT / BIOBANK NORWAY

2. Your background

3. Which description(s) fits the sample collection(s) you are referring to best? (Check all that apply)

- [ ] Population-based biobank (prospective population-based cohort)
- [ ] Prospective disease-oriented biobank
- [ ] Biobank or tissue archive containing tissue/specimens leftover from health care interventions (‘residual use’)
- [ ] Biobank containing tissue/specimens leftover from specific research projects (e.g. clinical trials)
- [ ] Genetic biobank
- [ ] Other (please specify)

4. Does the biobank handle and/or contain genetic data or samples of isolated DNA, RNA?

- ○ Yes
- ○ No
- ○ I don't know

5. This biobank is financed by

- ○ Public (national or regional) investments
- ○ Private investments
- ○ Both, public and private investments
- ○ I don't know

6. What is your main role related to biobanks?

- ○ Clinician
- ○ Clinician/Researcher
- ○ Health care assistant/Nurse
- ○ Genetic counsellor
- ○ ELSI consultant
- ○ Researcher
- ○ Administrative staff
- ○ Project manager
- ○ Director/CEO/Manager

Other (please specify)

7. In which country is your biobank/research facility located?

BBMRI-ERIC / COST ACTION CHIP ME / RD-CONNECT / IMI DO-IT / BIOBANK NORWAY

3. Informed Consent (from here on: IC)

Informed consent (IC) can refer to either a) a process or dialogue, or b) a more formal procedure involving provision of information and consent options. For the purpose of this survey, please consider both understandings of informed consent andrefer to the most recent IC process you have used. It might be helpful to have a copy of the IC form at hand as you answer the questions.

8. Is the informed consent sheet you are referring to publicly available (e.g. on a website)?

- Yes
- No
- I don't know

9. What information <u>is provided</u> to participants/donors in the informed consent procedure? Please refer to the most recent IC:

	**Is** provided in the IC	**Is not** provided in the IC	I don't know
General info about the biobank and who is responsible for the IC procedure	☐	☐	☐
Contact details of the biobank	☐	☐	☐
The purpose and (future) objectives of the associated research	☐	☐	☐
Details about research conducted through the biobank (e.g. via an online tool)	☐	☐	☐
Possibility for the participant/donor to be recontacted for additional data/samples	☐	☐	☐
Possibility of returning individual research results	☐	☐	☐
Linkage of data/samples with data from other sources (e.g. registries, national statistics, electronic health records, other research biobanks, non-health care related data,…)	☐	☐	☐
Sharing data/samples with other non-commercial research partners	☐	☐	☐
Sharing data/samples with commercial and/or health industry partners	☐	☐	☐
Sharing data/samples with parties in other EU countries	☐	☐	☐
Sharing data/samples with parties in non-EU countries	☐	☐	☐
Expected storage period for data/samples	☐	☐	☐
The right to withdraw IC at any time and what happens to data and samples afterwards	☐	☐	☐
Other rights of participants, e.g. right to access data or right to know how data is processed	☐	☐	☐
The right to lodge a complaint with a supervisory authority (e.g. an ethics commission or data protection officer), including contact info	☐	☐	☐

Other (please specify)

10. In your opinion, what information <u>should be provided</u> to participants/donors in the IC?

	**Should be provided** in the IC	**It is not essential** for the IC	I don't know
The purpose and (future) objectives of the associated research	☐	☐	☐
Possibility of re-contact by researchers for additional data/sampling	☐	☐	☐
Possibility of returning individual research results	☐	☐	☐
Sharing data/samples with other non-commercial research partners	☐	☐	☐
Sharing data/samples with commercial and/or health industry partners	☐	☐	☐
Sharing data/samples with parties in other EU countries	☐	☐	☐
Sharing data/samples with parties in non-EU countries	☐	☐	☐

Other (please specify)

BBMRI-ERIC / COST ACTION CHIP ME / RD-CONNECT / IMI DO-IT / BIOBANK NORWAY

4. Informed Consent (from here on: IC)

11. Which sample and data use <u>is currently covered</u> by the informed consent in the biobank you are referring to?

- ○ Data and samples will only be used for the study for which the participant gave consent
- ○ Data and samples will be used for multiple research projects without re-consent
- ○ Participants/donors can choose their consent preferences about what they want to be involved in (online or paper-based)
- ○ Data can be used without consent because of a statutory exemption
- ○ I don’t know

12. In your opinion, how should re-use be practiced in biobanks?

- ◯ Data and samples will only be used for the study for which the participant gave consent
- ◯ Data and samples should be used for multiple research projects without re-consent
- ◯ Participants/donors should choose their consent preferences about what they want to be involved in (online or paper-based)
- ◯ Data and samples could be used without consent because they are anonymised
- ◯ I don’t know
- ◯ Other (please specify)

13. In your opinion, when do you think re-consent is required?

- ◯ If data/samples are to be used for a study in a different field
- ◯ If the field changes
- ◯ If new technology requires a change in practices
- ◯ I don’t think re-consent is necessary
- ◯ I don't know
- ◯ Other (please specify)

14. In your opinion, besides the information given at the initial IC, how should participants/donors be further informed about sample and data use?

- ◯ I see no need for further information for participants/donors who participated in biobanks
- ◯ Biobanks should inform participants/donors about research activities performed with samples and data (e.g. password restricted online information, newsletters or reports)
- ◯ Participants/donors should be able to contact biobanking employees on an individual basis for information about the kinds of research that has been conducted with their personal samples and data (e.g. helpdesk)
- ◯ Participants/donors should be able to retrieve individualized information any time about the kinds of research that has been conducted with their personal samples and data (e.g. online platform or interface)
- ◯ I don't know
- ◯ Other (please specify)

15. Does the current IC cover the possibility to link data and samples with data from other sources (databases, registries, national statistics, electronic health records, other research biobanks, non-healthcare related data,…)

- ◯ Yes
- ◯ No
- ◯ I don't know

16. Overall, how would you evaluate the IC you are currently using?

- ◯ I think it is sufficient
- ◯ I think it would benefit from some improvements
- ◯ I think it would benefit from major changes

If you think that it would benefit from some improvement/changes, please specify which aspects:

17. If you were seeking information on how to design or improve your IC, where would you search for it? (Multiple answers possible)

- ☐ Information from within my working environment or ask colleagues
- ☐ The internet
- ☐ Ethical-legal guidance by professional information centres such as help desks for ethical and legal issues
- ☐ National guidance or standards for IC and/or corresponding templates
- ☐ International guidance or standards for IC and/or corresponding templates
- ☐ I don't know
- ☐ Other (please specify)

18. Which form/s of professional ethical and legal support in relation to the IC would you appreciate, if any? (Multiple answers possible)

- [ ] Help desk
- [ ] Training
- [ ] Web-based support such as interactive tools
- [ ] Online information such as a web page
- [ ] Templates that follow national or international standards for IC
- [ ] Training or support are not necessary
- [ ] I already have sufficient support
- [ ] I don't know
- [ ] Other (please specify)

5. Participant Involvement

The growing importance of biobanking is accompanied by debates on whether, and the extent to which, participants/donors should be involved in biobanks.

19. In which ways are biobank participants/donors involved in activities of the biobank? (Multiple answers possible)

- [ ] Information meetings such as open days, public meetings, science days
- [ ] Focus groups or workshops to discuss specific aspects of the research
- [ ] Surveys to collect data about the participants’ perspectives on specific topics
- [ ] Open forums such as online, web-based participant forums
- [ ] No specific engagement activities in place
- [ ] I don't know
- [ ] Other (please specify)

20. What obstacles for participant/donor involvement have you encountered in relation to your biobanking activities? (Select up to 3 most prominent obstacles)

- [ ] No interest in involving participants
- [ ] Too little qualified staff
- [ ] Too little financial resources
- [ ] Too little time resources
- [ ] Lack of information about findings gained by samples and data
- [ ] No appropriate IT solutions for providing information
- [ ] Complex research details are difficult to communicate
- [ ] No obstacles encountered
- [ ] I don't know
- [ ] Other (please specify)

BBMRI-ERIC / COST ACTION CHIP ME / RD-CONNECT / IMI DO-IT / BIOBANK NORWAY

6. Public Private Partnerships (commercial uses)

Publicly funded biobanks are increasingly encouraged to develop partnerships with the health industry such as pharmaceutical and biotechnology companies.

21. Does your biobank collaborate with health industry partners (such as pharmaceutical and biotechnology companies)?

- ○ Yes
- ○ No
- ○ Not yet, but we plan to develop collaborations with stakeholders from the health industry
- ○ No, and we do not plan to do so
- ○ I don’t know

22. In your opinion, how should biobanks inform their research participants/donors about potential or existing collaborations with stakeholders from the health industry?

- ◯ Biobanks should inform participants once such collaborations takes place (e.g. newsletter or web page)
- ◯ Biobanks should ask for participants' consent for each collaboration with the health industry
- ◯ Biobanks should explicitly ask for participants' broad consent for collaborations with the health industry at the time of recruitment
- ◯ Biobanks do not need to inform participants about collaborations with the health industry
- ◯ Other (please specify)

23. In your opinion, what are possible benefits when a biobank establishes collaborations with stakeholders from the health industry? (Please select up to 3 options or the last one)

- ☐ Increased knowledge on diseases and treatment
- ☐ Development of drugs and therapies
- ☐ Creation of new jobs
- ☐ Increased incomes for the biobank
- ☐ Personal benefits for the biobank participants, such as access to specific drugs
- ☐ Facilitate better research
- ☐ None of these outcomes are important

24. In your opinion, which risks are likely when a biobank establishes collaborations with stakeholders from the health industry? (Please select up to 3 options, or the last one)

- ☐ Industrial partner determines the direction or focus of research
- ☐ Industrial partner influences research results
- ☐ Focus on profit rather than public health requirements
- ☐ Industrial partners earning money while the biobank gets nothing equal in return
- ☐ The biobank becomes dependent on private funding
- ☐ None of these outcomes are likely

25. In your opinion, what is important to facilitate good collaborations between biobanks and stakeholders from the health industry? (Please select the 3 most important)

- [ ] Solid contracts describing the responsibilities of the partners
- [ ] Partners in the collaboration should share risks as fairly as possible
- [ ] Partners in the collaboration should share data and samples as fairly as possible
- [ ] Partners in the collaboration should share benefits as fairly as possible
- [ ] Both parties should be aware of the details of the collaboration
- [ ] Information about the details of the collaboration should be publicly available
- [ ] Other (please specify)

7. Thank you!

Thank you for participating in our survey!

We would be grateful if we could contact you directly for further research purposes. If you agree please provide your contact details here. You can also contact us by sending an email to: contact@bbmri-eric.eu
